# Anatomical Variation of the Pudendal Nerve and Related Structures

**DOI:** 10.1016/j.euros.2026.01.015

**Published:** 2026-02-09

**Authors:** Junjie Yang, Katie E. Webb, Emma V. Carrington, Emma M. Cullen, Alex Digesu, Karel Everaert, Ahmed Ibrahim, Harriet Kemp, Alison Mears, Jalesh N. Panicker, Marcus J. Drake

**Affiliations:** a Department of Surgery and Cancer, Imperial College London, London, UK; b Department of Physiotherapy, Imperial College Healthcare Trust, London, UK; c Department of Colorectal Surgery, Imperial College Healthcare Trust, London, UK; d Department of Gynaecology, Imperial College Healthcare Trust, London, UK; e Department of Urology, Ghent University Hospital, Ghent, Belgium; f Department of Neurosurgery, Imperial College Healthcare Trust, London, UK; g Division of Anaesthetics, Pain Medicine and Intensive Care, Imperial College, London, UK; h Department of Sexual Health, Imperial College Healthcare NHS Trust, London, UK; i Department of Uro-Neurology, The National Hospital for Neurology and Neurosurgery, London, UK; j UCL Queen Square Institute of Neurology, University College London, London, UK; k Department of Urology, Imperial College Healthcare Trust, London, UK

**Keywords:** Anatomical variation, Nerve entrapment, Pudendal nerve

## Abstract

**Background and objective:**

The pudendal nerve (PN) typically arises from sacral roots S2–S4 and gives rise to three main branches: inferior rectal, perineal, and dorsal genital nerves. However, conditions such as pudendal neuralgia and persistent genital arousal disorder exhibit great variability in clinical course and therapeutic responses. Anatomical variation of the PN may contribute to this variability by placing the nerve or its branches in vulnerable positions that lead to compression or traction. This scoping review examined PN anatomical variations to gain a better understanding of their role in pathophysiology and clinical outcomes.

**Methods:**

A scoping review was conducted according to the Preferred Reporting of Items for Systematic Reviews and Meta-Analyses-Extension for Scoping Reviews (PRISMA-ScR) guidelines to answer the following research question: What are the anatomical variations of the PN and related structures along its route? Searches were conducted in the MEDLINE and EMBASE databases, with manual screening. Studies on human anatomical investigations of PN variations, regardless of method, were included.

**Key findings and limitations:**

The review revealed substantial anatomical diversity in nerve roots, trunk, branches, and related structures, for which detailed schematic illustrations were developed. Limitations include methodological heterogeneity across studies, the predominance of elderly cadaver specimens, and lack of formal quality assessment.

**Conclusions and clinical implications:**

Anatomical variation is a key factor in the development and persistence of PN-related conditions. An understanding of this variability is critical for diagnosis, surgical planning, and effective management. This review challenges assumptions of “typical anatomy” and offers context for refinement of decompression techniques and therapeutic strategies.

**Patient summary:**

Our study shows that one of the nerves in the pelvis, called the pudendal nerve, varies between individuals. This could explain why standard treatments do not work in some patients and could help doctors to better understand these conditions.

## Take Home Message

Anatomical variation of the pudendal nerve is complex and clinically significant. Recognition of these variants is important for improving diagnosis, surgical planning, and treatment outcomes in conditions such as pudendal neuralgia and persistent genital arousal disorder.

## 1 Introduction

**Fig. 1:**
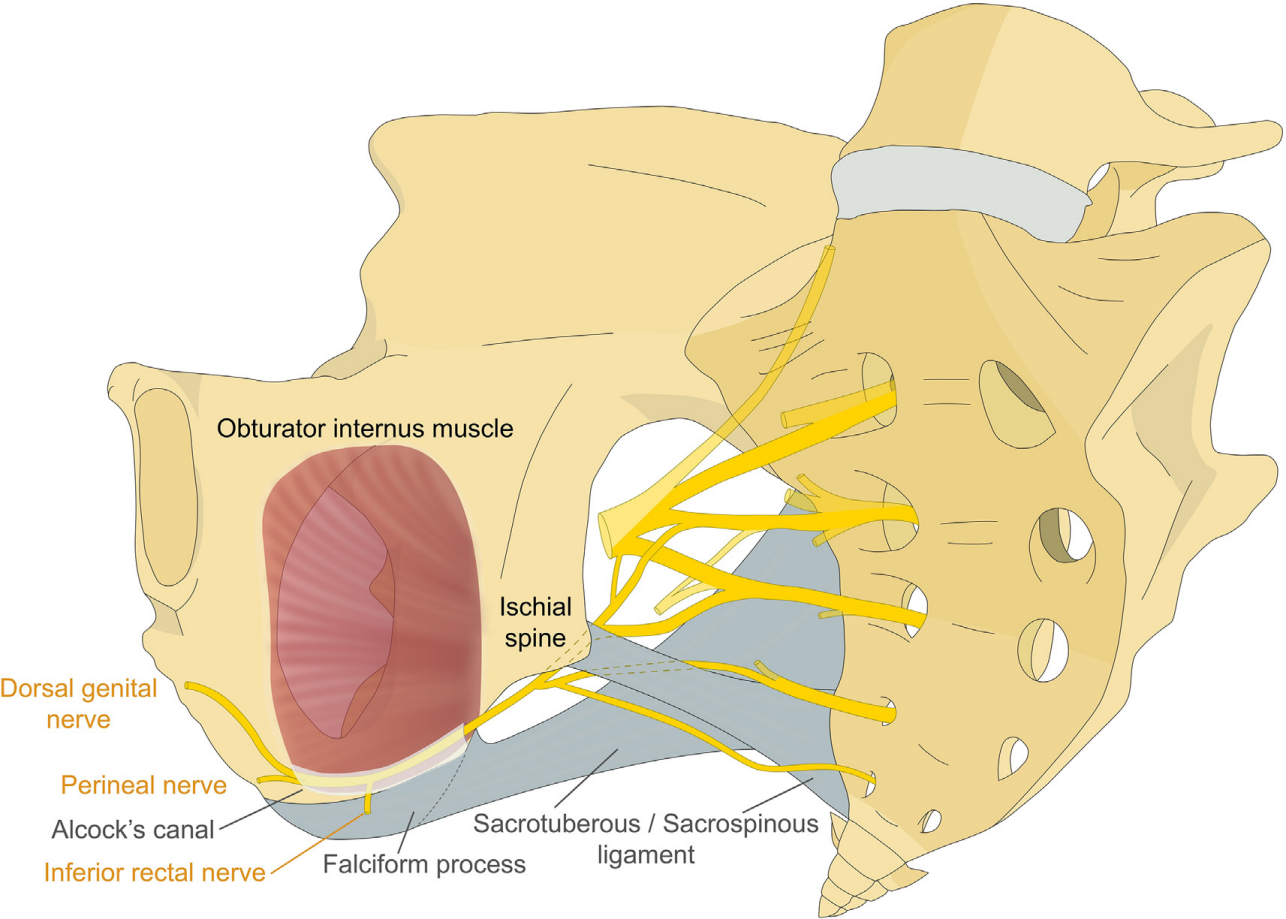
General model of the anatomical course of the pudendal nerve.

The pudendal nerve (PN) provides sensory, motor, and autonomic innervation to the distal anal canal, urethra, perineum, and genitals. It is generally considered that the PN arises from the ventral rami of the sacral S2, S3, and S4 roots in the sacral plexus, and passes between the piriformis and ischiococcygeus muscles (Fig. 1). The PN exits the pelvis through the greater sciatic foramen and crosses between the sacrospinous ligament (SSL) and sacrotuberous ligament (STL) before re-entering the pelvis through the lesser sciatic foramen. With the internal pudendal vessels, the PN traverses the pudendal canal (Alcock’s canal) in the fascia of the obturator internus muscle at the inferior margin of the obturator foramen, along the lateral boundary of the ischiorectal fossa. Within the canal, the PN divides into the inferior rectal nerve (IRN), terminal perineal nerve (PrN), and dorsal genital nerve (DGN; dorsal nerve of the penis or clitoris, DNP/C).

The PN crosses multiple anatomical regions (pelvic, gluteal, perianal/perineal, and pudendal regions) and is prone to entrapment in each of these, depending on aetiology. Variation within these regions can influence symptom presentation, treatment strategies, and outcomes. Entrapment at the SSL or pudendal canal without extensive fibrosis is often manageable, but peripheral lesions (eg, at the urogenital diaphragm) may cause severe fibrosis. Understanding PN anatomy is critical for clinical examination and surgery to reduce the risk of iatrogenic injury, entrapment, and complications such as pudendal neuralgia, voiding dysfunction, or anorectal symptoms.

PN entrapment (PNE) can cause neuralgia, persistent genital arousal disorder/genitopelvic dysaesthesia (PGAD/GPD), and sensory loss. The phenotype can include variability in the extent of local, regional, and generalised factors [1], [2]. PGAD/GPD involves intrusive, distressing genital arousal sensations, often with dysaesthesias (eg, tingling, burning, pain) and without sexual desire [3], [4]. Although the pathophysiology spans five possible levels from the genital organs to the brain, some cases present with well-localised pudendal-territory symptoms, which suggests discrete nerve pathology. A PN block is often administered, especially in pudendal neuralgia, for diagnostic and therapeutic purposes [1], [5]. However, results are inconsistent and unreliable. This may result from difficulty in infiltrating the nerve accurately in deep-sited locations, but could also suggest variation in anatomical aspects between patients. Pudendal neuralgia and PGAD/GPD are rare conditions, but they have a severe effect on quality of life and are often refractory to treatment, which can result in high morbidity and socioeconomic and health care–associated costs [1], [3].

Given the potential role of PN anatomical variations in these conditions, accurate mapping of the PN and its surrounding structures can improve diagnostics, treatments, and surgical outcomes. Here we report an in-depth scoping review of anatomical variants of PN distribution. Variants in the related structures (ligaments, pudendal canal) were also evaluated, as PNE or equivalent changes are likely to result from factors extrinsic to the PN.

## 2 Methods

### 2.1 Research question and aim

This scoping review addressed the following research question: What are the anatomical variations of the PN and related structures along its nerve route? The objectives were to: (1) document PN anatomical variations between sacral foramina and terminal branches; and (2) develop detailed visual representations of PN anatomical variations.

The review followed Preferred Reporting of Items for Systematic Reviews and Meta-Analyses-Extension for Scoping Reviews (PRISMA-ScR) and Joanna Briggs Institute Guidance for Scoping Reviews [6], and was registered with the Open Science Framework.

### 2.2 Eligibility criteria

The inclusion criteria were studies on humans of any age using anatomical research methodology (eg, cadaver studies, magnetic resonance imaging [MRI]) with a focus on anatomical variations of PN or related structures that were published as full-text articles in a peer-reviewed journal. There was no limitation on year of publication.

The exclusion criteria were: animal-only populations; review articles, narrative reviews, or book chapters; publications from non–peer-reviewed sources (including StatPearls); publications in languages other than English; publications with no full text available; studies including anatomical variation in terminal branches without reference to the main trunk; and studies focused solely on surgical approaches.

### 2.3 Information sources

A systematic search was conducted in the MEDLINE and EMBASE (via Ovid) databases, which was supplemented by manual screening of reference lists and related literature.

### 2.4 Search

The final search strategy combined relevant terms related to PN anatomical structures and variations, using adjacency operators to identify papers specifically focused on PN anatomy:

1. ((pudendal nerve* or inferior rectal nerve* or perineal nerve* or (Dorsal nerve adj3 clitor$) or (Dorsal nerve adj3 peni$)) adj8 (anatom* or locat* or varia*)).
2. limit 1 to (English language and humans).

The latest search was conducted on January 24, 2025. Results were exported to Covidence for screening.

### 2.5 Selection of sources of evidence

The study selection process involved two stages. One reviewer (J.Y.) first screened all titles and abstracts to identify potentially relevant studies. Two reviewers (J.Y. and K.E.W.) then independently assessed full-text articles against the eligibility criteria. Disagreements were resolved via discussion, and a third reviewer (M.J.D.) was consulted when needed.

The following data were then extracted from each study: publication characteristics; study design and objectives; sample characteristics; methodological details; anatomical findings; and surgical considerations.

### 2.6 Synthesis of results

Data were grouped by the type of anatomical variation mentioned, and quantitative data were derived descriptively. Schematic diagrams were developed on Adobe Illustrator for a visual demonstration of PN anatomical variations.

## 3 Results

### 3.1 Sources of evidence

**Fig. 2:**
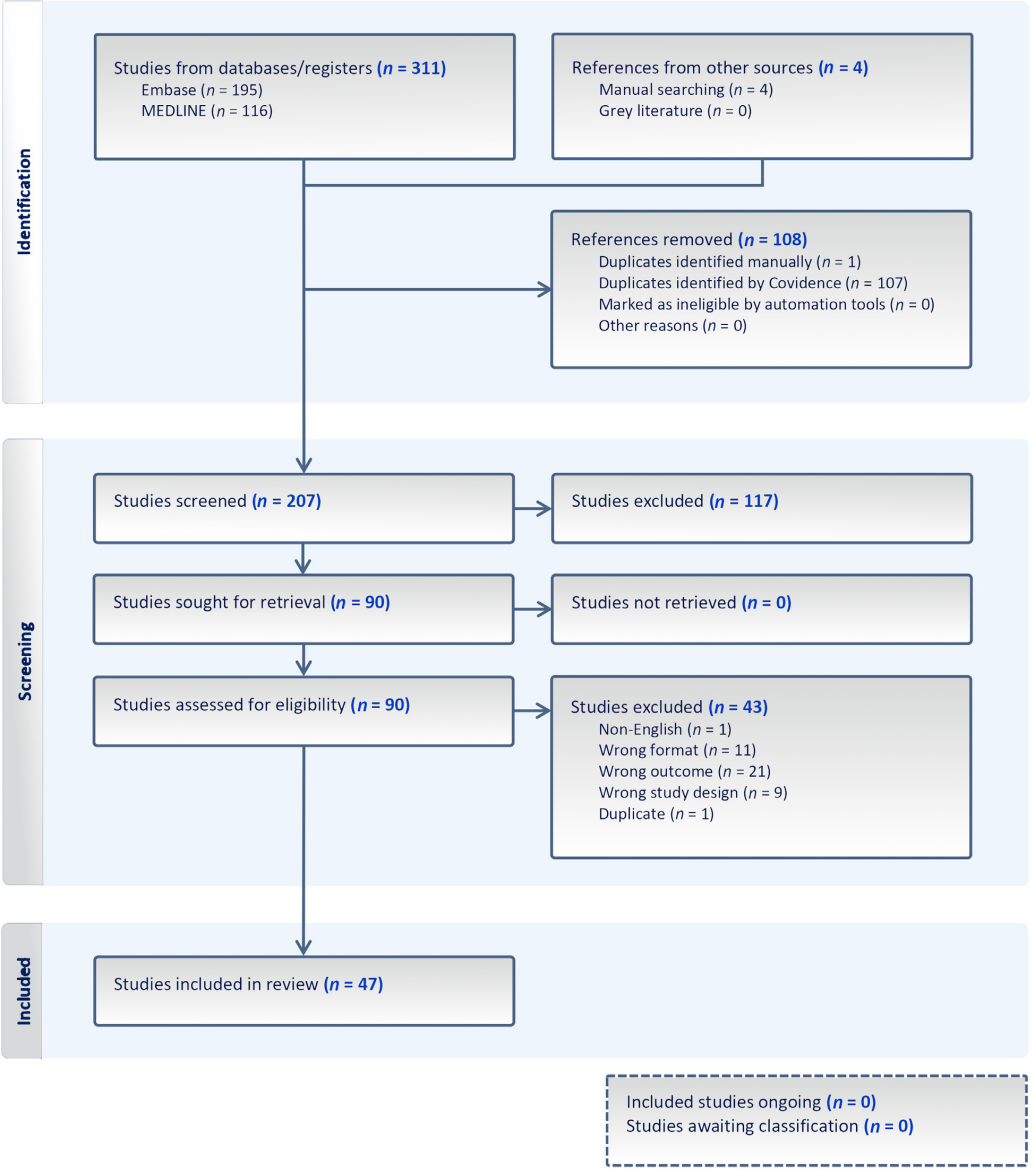
Preferred Reporting Items for Systematic Reviews and Meta-Analyses (PRISMA) flow diagram.

A total of 47 studies were included: 39 cadaver dissection studies, four mixed studies combining cadaver dissection with imaging (three MRI, one computed tomography), two surgical studies on patients, and one MRI study (Fig. 2). Five studies examined human foetuses. An overview of all the studies included is provided in the Supplementary material.

### 3.2 PN roots of origin

**Fig. 3:**
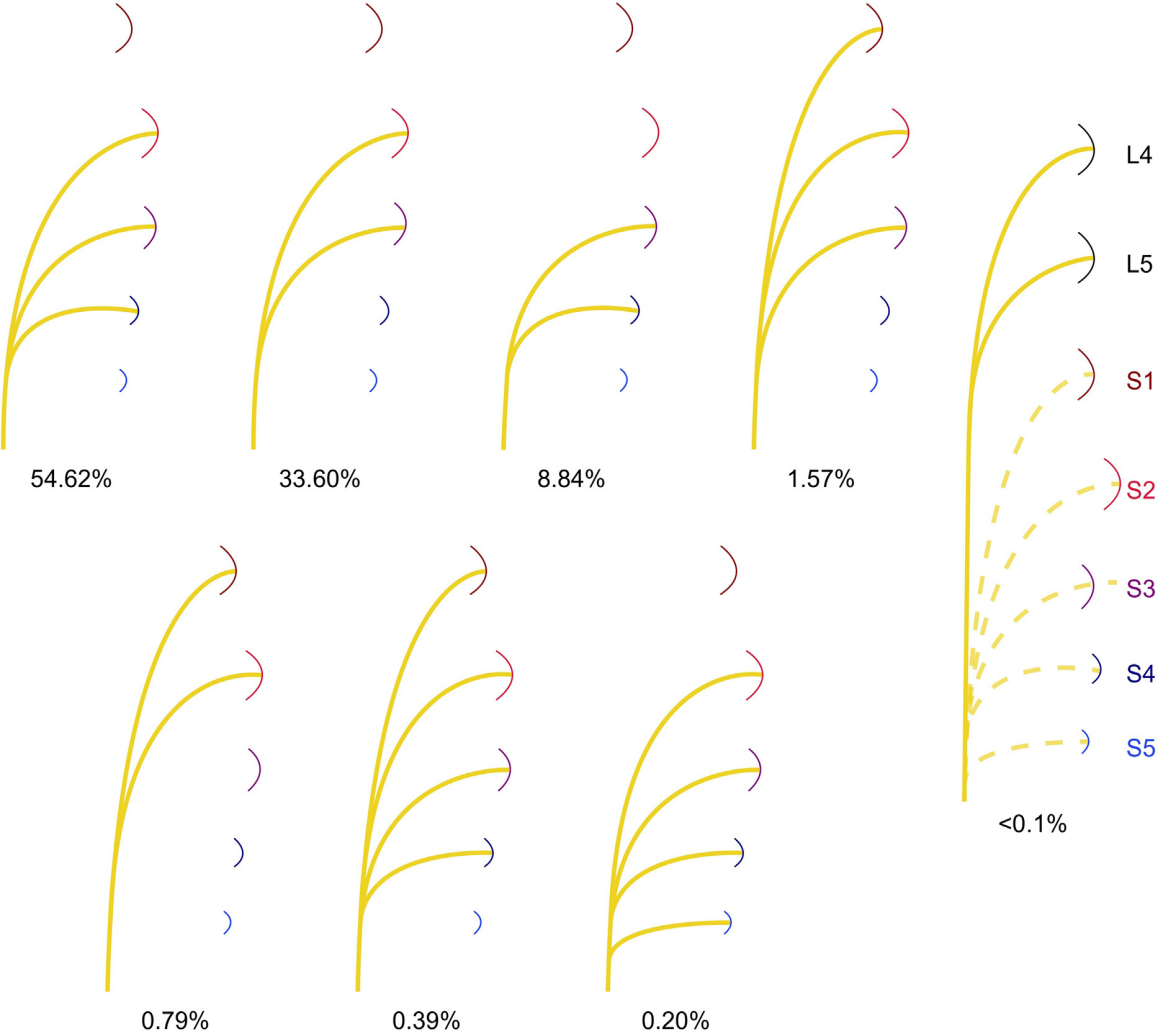
Origin roots of the pudendal nerve. Different combinations of sacral-root contributions in the formation of the pudendal nerve are illustrated, showing S1–S5 except for the right-hand image (marked L4–S5). Prevalence is summarised according to a combined analysis of 509 samples. The most common configuration was S2–S4, followed by S2–S3, S3–S4, S1–S3, S1–S2, S1–S4, and S2–S5. In addition, rare case studies reported contributions from lumbar roots L4 and L5.

Analysis of 18 studies revealed considerable variability in PN root contributions. A combined analysis of 509 specimens [7], [8], [9], [10], [11], [12], [13], [14], [15], [16], [17] identified the S2–4 pattern in 54.62% of cases, with the others set out in Fig. 3. A contribution of L4/L5 nerve roots in the DNP was reported in a case study [18].

Roots of PN origin have been categorised into S1–S2 dominant (40%) and S3–S4 dominant (60%) patterns [19]. In the S3–S4 dominant group, S3 was the predominant contributor in 25% of cases, while S4 was dominant in 8.33%. This study also found S2 participation in 85% of PN formations. Another study reported: S1 (6%), S2 (89%), S3 (97%), and S4 (74%) participation [7].

### 3.3 Main PN nerve trunk

A combined analysis of 645 specimens revealed four distinct trunk configurations. A single trunk was observed in 47.60% [7], [9], [15], [20], [21], [22], [23], [24], [25] and multiple in 52.40% of cases. Further analysis of multiple trunk patterns in 619 samples identified double-trunk patterns in 38.93% [7], [9], [20], [21], [22], [23], [24], [25], a triple trunk in 12.76%, and a quadruple-trunk pattern in 1.29% of cases.

**Fig. 4:**
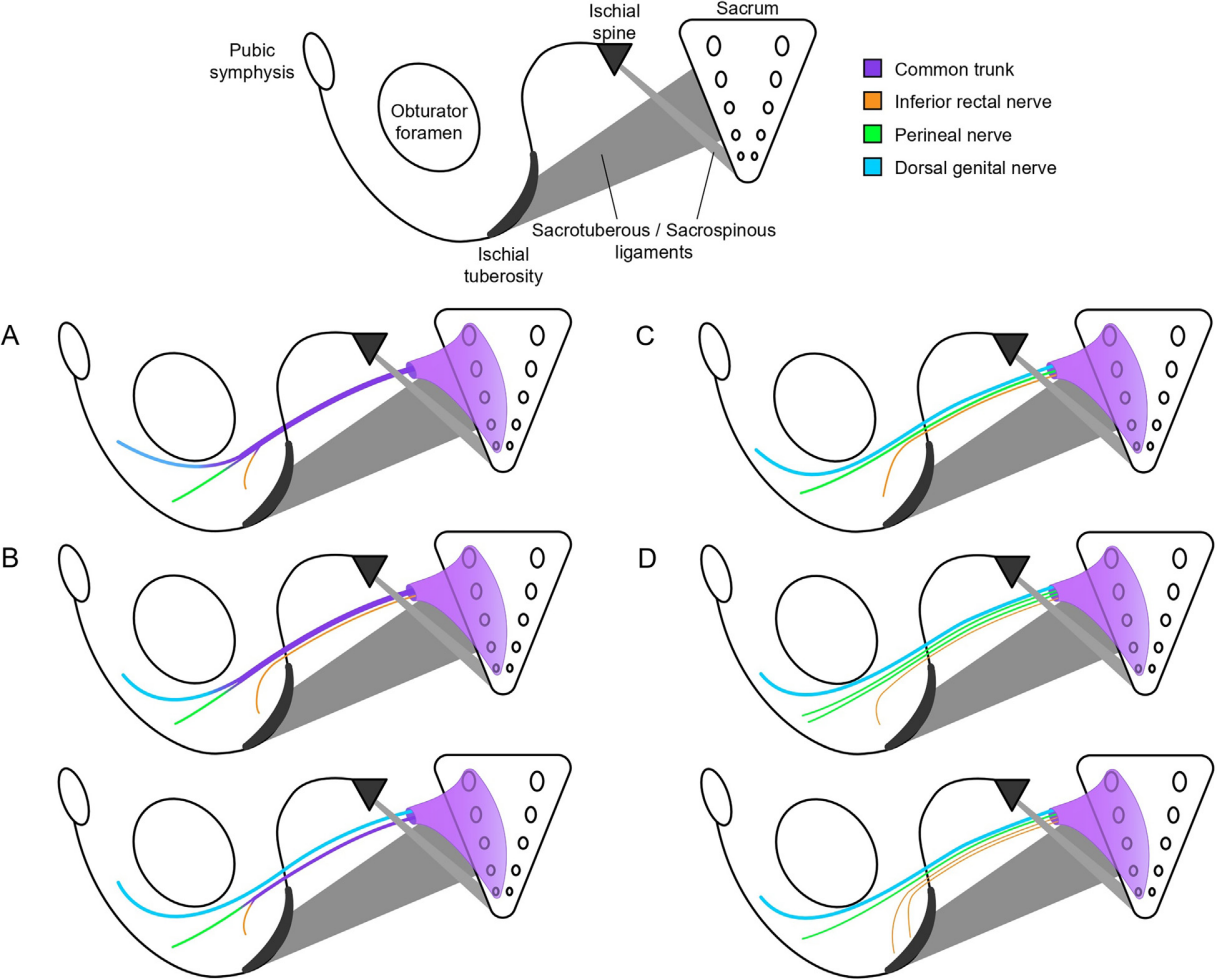
Trunk configurations of the pudendal nerve. Schematic representations show branching patterns of the pudendal nerve near the ischial spine. (A) Single-trunk pattern; the dorsal genital nerve branches first in some cases. (B) Double-trunk pattern, with rectopudendal or pudendogenital division. (C) Triple-trunk pattern, with separate rectal, perineal, and genital branches. (D) Quadruple-trunk variants, including duplicated rectal or perineal branches.
• Double trunk: classified by a terminal trunk-organisation pattern (Fig. 4B).
  o Type II: a rectopudendal pattern in which the pudendal trunk is 1.5 times larger than the rectal trunk, which is further subdivided into:
    • Type IIa: with the IRN branch piercing through the SSL; and
    • Type IIb: without the IRN branch piercing through the SSL.
  o Type III: a pudendogenito pattern.
• Triple trunk: type IV, with a recto-perineo-genito pattern (Fig. 4C).
• Quadruple trunk: type V, with a recto-perineo-perineo-genito or recto-recto-perineo-genito pattern (Fig. 4D).

A detailed combined classification of branching [7], [9], [22], [23], [24], [25], [26] comprised the following patterns:

• Single trunk: type I, a classical pattern with division before or within the pudendal canal. This pattern is further categorised according to the relative location of the branch [26] (Fig. 4A):
  o Type Ia, in which the IRN branch is proximal to the DGN (6/11); and
  o Type Ib, in which the DGN branch is proximal to the IRN (5/11).

Further complex trunk combinations have also been documented [27]. In addition, a two-cord formation in the sacral plexus has been described, with the upper cord formed by S2 (or S1) and the lower cord formed by S3-4 (or S5), which joined to form a single PN trunk [12], [28].

### 3.4 Ligament relationships

The PN typically forms from the union of two cords above the SSL, but in some cases it forms behind it [28]. Direct PN or branch penetration into ligaments was reported in nine studies, with nerve trunks piercing the SSL and/or STL [9], [11], [13], [18], [21], [25], [29], [30], [31]. Branch-specific (IRN) penetration of the SSL was also reported [9], [11], [13], [31]. Pirro et al [25] found PN penetration of the SSL in 32/40 and of the STL in two of 40 cases, while Ploteau et al [30] found seven cases of penetration (four STL, three SSL) in a cohort of 145 patients.

Ligament changes included thickened ligaments in 39% of cases, with the STL forming a fibrous sheath in 8% [25]. Sclerotic changes were documented in 25% of cases (five of 20), all in individuals aged >70 yr, with thickened PN showing evident macroscopic changes within the range of the SSL in 20% of cases (four of 20), all with calcifications of the SSL [19]. In an age-related unilateral STL ossification case, there was spiralling and an irregular bony protuberance that affected nerve mobility [32]. In some cases the STL split into a fan-like formation. Alternatively, the SSL split into three bands, with one band almost transecting the PN, which thus represented a potential compression point [31].

The interligamentous space was described as triangular in shape [33], [34] with a mean size of 6.7 ± 1.7 mm (range 4–10 mm), but was <5 mm in 15% of cases [19]. Interligamentous bands arise that are associated with perineural compression (*p* = 0.033), often with bilateral tendency [35], including:

• Delayed separation of the SSL and STL distal to the sacrococcygeal junction (*p* < 0.0001);
• Close apposition of the SSL and STL (*p* < 0.0001); and
• Pubococcygeal muscle contributions to the SSL (*p* = 0.014).

The falciform process (FP) is a sickle-shaped extension of the STL towards the ischial ramus, which terminates at the obturator fascia (69%), or fuses with the fascia and continues to the ischiorectal fossa (18%), which thickens the fascial sheath and limits free space in the pudendal canal [36]. The FP was noted as a fascial extension of ligaments where the IRN branches off [10]. Sometimes the FP was proximal and thickened unilaterally (*p* < 0.0001) [35].

The interligamentous space is a compression zone [27], [30]. The PN was compressed by the inferior margin of the SSL in 40% of cases (eight of 20), in each case running 2–5 mm from the SSL attachment to the IS [19]. Limited nerve mobility contributed to entrapment between the ligaments. The PN was fixed by connective tissue to the dorsal surface of the SSL in all specimens (26/26), becoming freely mobile only after the SSL was dissected from the IS and surrounding connective tissue [15]. A connective tissue sheath around the neurovascular bundle at the pudendal canal entrance represented another potential entrapment site [27].

### 3.5 Ischial spine

Two studies found that the PN passed medially to the ischial spine (IS) in 77–80% of cases, with posterior and lateral passage in the remainder [12], [37]. The IRN passed medially in 62% [38].

The IS shows morphological variations that include broad and flat, broad and flared, or sharp and narrow forms [33]. Probability of compression at or above the IS was identified in 34.7% of female and 36.4% of male samples [35]. A clinical anomaly was noted where the PN passed 2 cm lateral to its typical position, which resulted in compression against bone by the SSL [31].

### 3.6 Pudendal canal

The pudendal (Alcock’s) canal is formed by the division of the obturator internus fascia [14], [39], with a triangular entrance formed by the obturator internus fascia, SSL, and FP, which suggests a possible compression site [27].

Nerve branching patterns within the canal varied, with the PN entering undivided in 80–90% of cases and the IRN originating independently from S3 or S4 without entering the canal in the remainder [13], [40]. The IRN exited by piercing the canal wall [12], [40], [41], [42], with restricted nerve mobility indicating fixed points that may facilitate entrapment [15]. This can be compounded by acquired characteristics, such as perineural vasculature changes (four of 263), thickening of the obturator fascia (two of 263), an enlarged obturator internus muscle (one of 263), and perineural fibrosis (one of 263) [35].

### 3.7 Inferior rectal nerve

The IRN origin patterns are “classic” (within or before pudendal canal), or “variant” (directly from sacral roots or the plexus in 38.01% of cases) [11], [12], [13], [33], [40], [43], [44]. Root-specific contributions to variant IRN included: S3 alone (16.7%), S4 alone (50%), and S3 and S4 (33.3%) [12]. Direct SSL penetration by the IRN was observed in 10–36% of cases (*n* = 187) [12], [31], [41], [42], with high occurrence of the variant pattern. This was associated with a communicating branch from the main pudendal trunk [44].

### 3.8 Perineal nerve

The classical PrN pattern is emergence within the pudendal canal [14], [43], but it can arise directly from the sacral plexus [27], [30]. The PrN pierces the pudendal canal wall and enters the ischiorectal fossa [28], [45]. Communicating branches with the sciatic nerve and IRN have been described [11], [18]. PrN branching patterns included bifurcation into superficial and deep branches [45], and three distinct fascicular branches [26]. The PrN typically innervated the urinary rhabdosphincter [46], but also other areas, such as puborectal and pubovaginal muscles [17].

### 3.9 Dorsal genital nerve

Studies revealed that the DGN (DNP/C) emerges from the sacral plexus or root directly, with one case study showing contributions from L4 and L5 nerve roots [18], [19], [27]. The DGN branched from the PN before entering the pudendal canal in 75% of cases, and within the canal in 25% [19]. The reported course was consistent across studies [14], [43]. After exiting the canal, the DGN typically traverses a groove on the inferior surface of the pubic bone—termed the sulcus nervi dorsalis penis/clitoridis—which is a region susceptible to compression [47]. This sulcus has been classified into three anatomical types according to its presence and length, and such structural variations may influence the susceptibility to DGN compression within the groove. Distally, the nerve passes through a tight osteofibrous canal formed in part by the inferior pubic ramus [48].

On the penile shaft, the DNP consists of two populations of axons: the dorsal midline trunk travels as a single trunk to the glans, and lateral branches (0–4 in number) pass ventrolaterally over the penile shaft with asymmetric variation [49]. Extensive branching within the glans formed a dense, three-dimensional network. Significant anatomical variation has been reported, with the DNP composed of two to six main branches, and two to five terminal branches entering the glans [50]. In 72.7% of cases, perforating branches passed from the inferior aspect of the DNP into the tunica albuginea.

The DNC is positioned closer to the bony frame of the pubis in females than the DNP is in males [37]. On average, the DNC was located 0.56 mm from the pubic symphysis in females of African ancestry and 0.15 mm in those of European ancestry.

### 3.10 Entrapment zones

Four primary compression sites were identified [27], [30], [31], [45], [51]:

1. Pelvic region (70%):
  o Infrapiriform foramen (lower part of the greater sciatic foramen).
  o Subpiriformis space.
  o Lesser sciatic foramen.
2. Interligamentous space between the SSL and STL (90%), and the SSL [19], [31].
3. Entrance of the pudendal canal at the FP (10%).
4. Within the pudendal canal (10–20%).

Multiple entrapment sites in the same patient were common [30]: six patients showed compression at sites 1 and 2, 33 patients (44 nerves) at sites 2 and 3, and eight patients (nine nerves) at sites 1 and 3.

### 3.11 Muscular variations

Considerable variation in the thickness of the obturator internus muscle has been observed, ranging from 11.0 to 28.4 mm (median 20.7 mm) [35]. Pathological changes in the obturator internus muscle included muscle hypertrophy in 7% [30] and muscle atrophy in 15% of cases [19], which are variations that could affect the diameter of the pudendal canal. The piriformis, related to the PN course as it exits and re-enters the pelvis, showed similar variability [35]. Pelvic-floor muscles likewise varied, with a median thickness of 2.9 mm (range 1.2–6.6 mm); the coccygeus showed the greatest variability (median 2.2 mm, range 0.7–12.0 mm) [35].

### 3.12 Neurovascular relationship

There was variability in PN-vascular relationships [12], [21], [25]. A medial PN position relative to the vessels was predominant, followed by lateral and complex (vessels between multiple PN trunks). Gruber et al [21] found that 15.5% of nerve components were positioned dorsal or ventral to the artery, rather than in the same plane. Tagliafico et al [52] noted consistent anterior positioning of the artery relative to the nerve. Reversal of the topographic relationship of pudendal vessels and the PN between the greater sciatic foramen and the pudendal canal indicates that the artery crosses the nerve [45]. Nerve compression by the pudendal artery occurred in 1.4% of cases [30].

### 3.13 Other features

Peng et al [35] identified a perineural canal—distinct from the pudendal canal—encasing the PN in a fascial and adipose sheath, with a median diameter of 4.0 mm (range 1.0–11.0).

An “inferior pubic ramus canal” was described as a 3-cm osteofibrous tunnel formed by the inferior pubic ramus, suspensory ligament of the penis, and ischiocavernous body [27], [48]. Fusiform pseudoneuromatous thickening (two of ten) indicated possible neural compression.

An “accessory rectal nerve” arose in all seven male cadavers examined in one study, branching from the PN posterior to the SSL and innervating perineal/perianal skin and the ventral levator ani surface [28]. Similarly, bilateral accessory PNs were also found, originating from S1–S3 and joining the PrN, DGN, and posterior femoral cutaneous nerve [29]. Moreover, an ischiorectal fossa nerve plexus was identified in 17.9% of cadavers, which consisted of interconnected branches of the IRN and PrN [43].

The PN formed an independent plexus in 55% of cases, separated from the sacral plexus by the “liminal/limitans nerve”, while in the remaining 45% of cases the PN emerged directly from the sacral plexus [7], [8]. This nerve formed from the bifurcation of ventral rami S2, S3, or S4 and served as a boundary between the PN and the sacral plexus. A perforating cutaneous nerve from the PN was observed in 33.3% of cases [53].

## 4 Discussion

Our aim was to identify structural risk factors affecting the PN and its branches. A systematic review revealed considerable anatomical variation not only in the nerve itself but also in surrounding structures that may influence PNE risk. These variations become more clinically significant with age-related changes such as fibrosis and calcification, which are common among older individuals. Such changes may contribute to conditions that include pudendal neuralgia, PGAD/GPD, and sensory loss [2], [3], [54]. Furthermore, PN blocks rely on infiltration of local anaesthetic and anti-inflammatory medication into the pudendal canal or close to the IS [51], [54]. While this approach assumes a consistent anatomy, the PN variability observed could explain inconsistent treatment responses [5]. These anatomical variations, their associated aetiologies, and corresponding surgical approaches vary significantly by location along the course (Table 1). Indeed, many variations are likely to have implications for the emergence of clinical problems affecting the PN or its branches, such as pudendal neuropathy and PGAD/PGD. Constriction, angulation, and transection could all affect nerve function. Acquired symptoms could result from calcification of ligaments or the presence of additional surgical problems (eg, pelvic organ prolapse) that cause tension in the nerves. Hence, some clinical presentations may be explained by an anatomical predisposition.

**Table 1:** PN anatomic variations: regional distribution, causative factors, and surgical approaches

	Proximal	Pudendal canal		Distal/peripheral
	(SSL/STL region	Proximal	Distal	
Common anatomic variations	PN/IRN piercing SSL; ligament thickening & calcification; narrow interligamentous space	PN enters undivided or divided; IRN independent origin; FP thickening; triangular canal entrance	IRN piercing canal wall; variable branching patterns; limited nerve mobility; fascial thickening	DGN proximity to pubic symphysis; tight osteofibrous canal; variable pubic sulcus anatomy
Typical aetiologies	Muscle hypertrophy; vascular compression; prolapse surgery; age-related fibrosis	Prolapse and reconstructive surgery; RP; arterial compression; obturator internus enlargement or inflammation	Midurethral tape (transobturator) placement; RP; mesh complications	Cycling trauma; use of midurethral tape (transvaginal); pubic diastasis; urethral surgery
Surgical approach	Posterior approach; TG decompression; RAB for prolapse cases	RAB; prolapse surgery modifications; image-guided interventions	Perineal approach; prolapse surgery with nerve mapping; sling revision procedures	Perineal; transgluteal; laparoscopic or robotic

DGN = dorsal genital nerve; FP = falciform process; IRN = inferior rectal nerve; PN = pudendal nerve; RAB = retroabdominal; RP = radical prostatectomy; TG = transgluteal; SSL = sacrospinous ligament; STL = sacrotuberous ligament.

Peripheral PN components are often overlooked despite their role in cycling-related neuropathy and postsurgical complications. Given the structure of the pubic sulcus and surrounding canal [48], the dorsal nerve is more likely to be compressed at the pubic bone margin than within the pudendal canal itself [47]. Ligament calcification and sclerosis may fix the PN position, which increases the risk of traction, especially in cases of pelvic organ prolapse. A hypertrophied obturator internus can also raise fascial pressure and thus lead to compression of the PN.

Despite anatomical variability, proximity of the PN to the dorsal surface of the SSL is a consistent finding. This proximity is critical in surgical procedures involving the SSL, with a risk of iatrogenic PN entrapment and compression. Knowledge of such relationships may reduce the intraoperative risk of PN damage in urogynaecological operations to treat urogenital prolapse involving fixation and suspension of the prolapsed vagina and/or cervix, and in safe dissection and section of the ligament during nerve decompression procedures.

Variations in the PN trunking and component nerves were also identified. The DGN was fairly consistent and may have lower anatomical risk than the other branches. However, in some women, the close proximity of the DGN to the pubic symphysis places it at potential risk of conditions such as pubic diastasis and pubic osteophytes [55]. DNP variation in men affects electrode placement in neurophysiological studies. Overall, there were many prevalent anatomical variations, along with rare but potentially important features, such as bands almost transecting the PN, PN compression by the SSL inferior margin, and a PN running 2 cm lateral to its expected course. Such features would be difficult to identify clinically. MRI is increasingly being used, and advanced quantitative techniques such as diffusion tensor imaging can assess microstructural changes in the PN [56]. Pelvic neurophysiological tests can be used to evaluate the PN trunk and its branches [57]. However, further studies are needed to determine whether these techniques can identify PN anatomical variants.

Entrapment typically occurs at the IS between the SSL and STL. Multiple decompression techniques exist (transvaginal, transperineal, transgluteal, and laparoscopic), but none fully addressed all entrapment sites. Anatomical variations might contribute to treatment failure and complications with each approach [58].

These findings have direct relevance to electrode placement and neuromodulation in PN neurophysiological studies and treatment. They also affect interventional treatments for various urogynaecological conditions, notably PN stimulation, that rely on standard landmarks such as the IS and SSL. Hence, anatomical variations documented in this review affect accurate electrode placement, notably nerve-trunk configurations and nerve-ligament relationships. Current surgical protocols use electrostimulation or neurophysiological monitoring to confirm appropriate nerve contact [59], [60], but they did not facilitate recognition of nerve or branch location.

Limitations of the review include the heterogeneity of the study methodologies and populations, and the lack of systematic evaluation of potential influences such as differences between the sexes, ethnic groups, and laterality. Lack of standardised measurement protocols and classification systems constrained synthesis and interpretation of results. In addition, inherent limitations in anatomical research contribute to potential bias, such as the preponderance of older people in cadaver studies, and limited access to detailed medical histories. Small sample sizes in many studies restrict statistical analysis. Most studies documented variations in isolation without exploring potential correlations, which represents a clinically important knowledge gap. Lastly, initial screening of titles and abstracts was conducted by a single reviewer, which may have introduced selection bias, and no formal assessment of the quality of the studies included was performed.

## 5 Conclusions

Our scoping review revealed that the PN and related structures show considerable anatomical variation. We found important differences in nerve origins, trunk patterns, and relationships with nearby ligaments and other structures. These variations may explain variations in treatment responses for patients with PN conditions.

  ***Author contributions***: Marcus J. Drake had full access to all the data in the study and takes responsibility for the integrity of the data and the accuracy of the data analysis.

  *Study concept and design*: Yang, Webb, Drake.

*Acquisition of data*: Yang, Webb.

*Analysis and interpretation of data*: Yang, Webb, Drake.

*Drafting of the manuscript*: Yang, Drake, Carrington, Digesu, Everaert, Mears, Panicker.

*Critical revision of the manuscript for important intellectual content*: Ibrahim, Kemp.

*Statistical analysis*: Cullen.

*Obtaining funding*: None.

*Administrative, technical, or material support*: Drake.

*Supervision*: Drake, Webb.

*Other*: None.

  ***Financial disclosures:*** Marcus J. Drake certifies that all conflicts of interest, including specific financial interests and relationships and affiliations relevant to the subject matter or materials discussed in the manuscript (eg, employment/affiliation, grants or funding, consultancies, honoraria, stock ownership or options, expert testimony, royalties, or patents filed, received, or pending), are the following: Jalesh N. Panicker reports research support from the UK National Institute of Health Research Biomedical Research Centres funding scheme. Marcus J. Drake reports a trustee role for the International Continence Society; research funding from the Rosetrees Trust and the National Institute of Health Research; and speaker fees from Astellas. The remaining authors have nothing to disclose.

  ***Funding/Support and role of the sponsor*:** None.

  ***Acknowledgments*:** We thank Rebecca Jones for support with the literature search.
