# Supplementary material for: Anatomical Variation of the Pudendal Nerve and Related Structures
Source: Eur Urol Open Sci. 2026 Feb 9;85:71–81. doi: 10.1016/j.euros.2026.01.015 (PMC12908042; doi:10.1016/j.euros.2026.01.015)
Supplement: Supplementary Data 1 [file mmc1.docx]

**Appendix A: Summary of included literatures**

| **Author & Year** | **Study setting** | **Population description** |
| --- | --- | --- |
| OzcivitErkan 2024 | Cadaver study | 5 Female cadavers; the most common cause of death was a cardiovascular event, with no previous pelvic surgery, fractured pelvic bones, or trauma |
| Peng 2024 | MRI study on patients | 136 samples (124F, 11M, 1 trans-female); Relevant clinical history was available for 82 patients with high rates of previous intervention including laparoscopy (76.8%), neuropathic pain prescription (75.6%) and pelvic floor physiotherapy (80.5%) |
| Jee 2015 | Cadaver study | Case study of 62 YO female cadavers (right gluteal region only) |
| Gruber 2001 | Cadaver study | 58 Cadavers (29F 29M) |
| KLINK 1953 | Cadaver study | 25 Adult female cadavers and 60 recently dead females |
| Olszewski 1982 | Cadaver study | 50 Formalin fixed fetuses (22 F 28 M) with crown-rump length range from 154 - 291 mm |
| Olszewski 1986 | Cadaver study | 100 human fetuses (50 F 50 M) with crown-rump length range from 110 - 290 mm 10 adults (gender not specified) |
| Sikorski 1987 | Cadaver study | 100 Cadavers (50F 50M) |
| Shafik 1995 | Cadaver study | 10 Mature neonate (6 F 4 M) and 10 adults (7 F 3 M) |
| Hollabaugh 1997 | Cadaver study | 12 Fresh male cadavers, with no chemical preservation |
| Shafik 1999 | Cadaver study | 12 Cadavers (7F 5M): 6 fully mature neonates and 6 adults |
| Schraffordt 2004 | Cadaver study | 16F 13M (one sample excluded, gender unknown)  28 Cadavers fixed in 2% formaldehyde, 2.7% phenol, 33% glycerin, 38% ethanol and water |
| Mahakkanukrauh 2005 | Cadaver study | 73 Pudendal nerves from 37 cadavers (16 F 21 M) |
| Pirro 2005 | Cadaver study | 15 Human adult cadaver (7 F 8 M) preserved in formalin (n=8) or Winckler's liquid (n=7) |
| Hruby 2005 | Cadaver study | 10 Formalin treated hemipelves (gender not specified) |
| Seizeur 2005 | Cadaver and CT imaging study | 8 Human cadavers (3 F 5 M) |
| Gustafson 2005 | Cadaver study | 7 Formalin fixed cadavers (6 F 1 M) |
| Achtari 2006 | Cadaver study | 10 Embalmed hemipelves (gender not specified) |
| Nayak 2006 | Cadaver study | Right gluteal region of a 45-year-old male cadaver |
| Grigorescu 2008 | Cadaver study | 17 Formaldehyde-fixed female cadavers |
| Lazarou 2008 | Cadaver study | 15 Formaldehyde-fixed female cadavers |
| Kocabiyik 2008 | Cadaver study | 25 Formalin-fixed, spontaneously aborted fetuses (15 F 10 M) |
| Prat-Pradal 2009 | Cadaver study | Fresh human cadavers (5 F 3 M) |
| Pirro 2009 | Cadaver study | Human adult cadavers preserved in formalin or Winckler's liquid (9 F 11 M) |
| Yi 2010 | Cadaver study | 74 YO Japanese female cadavers, died from ovarian carcinoma |
| Colebunders 2011 | Cadaver study | 5 Formalin-fixed cadavers (1F 4M) and 1 fresh male cadaver |
| Barbe 2011 | Cadaver study | 11 Formalin-fixed cadavers (5F 6M) |
| Montoya 2011 | Cadaver study | 14 Un-embalmed and 4 embalmed female cadavers |
| Matejcik 2012 | Cadaver study | 20 Fresh cadavers (gender not specified) |
| Tagliafico 2013 | Cadaver study and imaging study (MRI) | 3 Human cadavers (2F 1M) and 20 healthy volunteers (15F 5M) |
| Furtmuller 2014 | Cadaver study and imaging study (MRI) | 12 Formalin-fixed cadavers for dissection (8F 4M) 2 Fresh cadavers sample for 3-T MRI (1F 1M) |
| vanderWalt 2015 | Cadaver study | 71 Adult cadavers of both European and African ancestry (29F 42M) |
| Maldonado 2015 | Cadaver study | 13 Un-embalmed female cadavers |
| vanderWalt 2016 | Cadaver study | 29 Female cadavers, 13 of African and 16 of European ancestry |
| Konschake 2017 | Cadaver study | 31 Formaldehyde-phenol or alcohol-glycerin fixed cadavers (gender not specified) |
| NyangohTimoh 2017 | Cadaver study | 7 Female fetuses cadavers without maceration neither macroscopic abnormality on pathology examination, with a crown-rump length of 110 - 310mm |
| Ploteau 2017 | Surgical study on patient | 100 Patients underwent unilateral or bilateral nerve decompression (58F 42M) |
| Cvetanovich 2018 | Cadaver study | 6 Fresh-frozen male cadaveric hemi-pelvises |
| Choi 2020 | Cadaver study | Female cadaver case study (only right hemipelvis being used) |
| Shafarenko 2023 | Cadaver study | 6 Formalin-fixed cadavers (3F 3M) |
| Hanna 2024 | Cadaver study | 22 Formaldehyde fixed cadavers (11F 11M) |
| Antolak 2024 | Surgical image study | Using real-world data, study participants were drawn from a community-based registry of pudendal neuropathy patients diagnosed by the Center for Urologic and Pelvic Pain (CUPP) over the course of 17 years, ending in 2020. Most had failed multiple past treatments (gender not specified) |
| Shafik 1999 | Cadaver study | 26 cadavers: 10 stillborn and 16 adults (gender not specified) |
| Roberts 1973 | Cadaver study | 20 cadavers (gender not specified) |
| Loukas 2006 | Cadaver study | Adult cadavers fixed in formalin-phenol-alcohol, no evidence of previous surgical procedures or traumatic lesions to the perineal or gluteal regions (15F 35M) |
| Jiri 2006 | Cadaver and skeletal sample study | 12 formalin-fixed cadavers for neuroanatomical dissection (6F 6M);  386 skeletal specimens examined (118F 168M) |
| Claire 1998 | Cadaver and electrodiagnostic study | Total 28 specimens dissected: 22 for shaft innervation (38 dorsal nerves), 6 for glans;  10 healthy volunteers were recruited for electrodiagnostic test |
